# A feasibility study for the provision of electronic healthcare tools and services in areas of Greece, Cyprus and Italy

**DOI:** 10.1186/1475-925X-10-49

**Published:** 2011-06-07

**Authors:** Stavroula G Mougiakakou, Efthyvoulos Kyriacou, Kostas Perakis, Homer Papadopoulos, Aggelos Androulidakis, Georgios Konnis, Riccardo Tranfaglia, Leandro Pecchia, Umberto Bracale, Constantinos Pattichis, Dimitrios Koutsouris

**Affiliations:** 1 Institute of Communication and Computer Systems, National Technical University of Athens, Athens, Greece; 2 Department of Computer Science, University of Cyprus, Cyprus; 3 National Center for Scientific Research "Demokritos", Athens, Greece; 4 Department of Biomedical, Electronic and Telecommunication Engineering, University of Naples Federico II, Naples, Italy; 5 ARTORG Center for Biomedical Engineering Research, University of Bern, Bern, Switzerland; 6 Division of Endocrinology, Diabetes and Clinical Nutrition, University Hospital - Inselspital - University of Bern, Bern, Switzerland; 7 Department of Computer Science and Engineering, Frederick University, Lemesos, Cyprus; 8 Department of General, Vascular and Thoracic Surgery, University of Naples Federico II, Naples, Italy

## Abstract

**Background:**

Through this paper, we present the initial steps for the creation of an integrated platform for the provision of a series of eHealth tools and services to both citizens and travelers in isolated areas of thesoutheast Mediterranean, and on board ships travelling across it. The platform was created through an INTERREG IIIB ARCHIMED project called INTERMED.

**Methods:**

The support of primary healthcare, home care and the continuous education of physicians are the three major issues that the proposed platform is trying to facilitate. The proposed system is based on state-of-the-art telemedicine systems and is able to provide the following healthcare services: i) Telecollaboration and teleconsultation services between remotely located healthcare providers, ii) telemedicine services in emergencies, iii) home telecare services for "at risk" citizens such as the elderly and patients with chronic diseases, and iv) eLearning services for the continuous training through seminars of both healthcare personnel (physicians, nurses etc) and persons supporting "at risk" citizens.

These systems support data transmission over simple phone lines, internet connections, integrated services digital network/digital subscriber lines, satellite links, mobile networks (GPRS/3G), and wireless local area networks. The data corresponds, among others, to voice, vital biosignals, still medical images, video, and data used by eLearning applications. The proposed platform comprises several systems, each supporting different services. These were integrated using a common data storage and exchange scheme in order to achieve system interoperability in terms of software, language and national characteristics.

**Results:**

The platform has been installed and evaluated in different rural and urban sites in Greece, Cyprus and Italy. The evaluation was mainly related to technical issues and user satisfaction. The selected sites are, among others, rural health centers, ambulances, homes of "at-risk" citizens, and a ferry.

**Conclusions:**

The results proved the functionality and utilization of the platform in various rural places in Greece, Cyprus and Italy. However, further actions are needed to enable the local healthcare systems and the different population groups to be familiarized with, and use in their everyday lives, mature technological solutions for the provision of healthcare services.

## Background

The southern and eastern Mediterranean countries extend over South Europe, Asia and North Africa- a geographically extended region that cannot be considered as a homogeneous group. The region is characterized by socio-demographic heterogeneities, expanded inner space and isolated areas; facts which hinder - if not prohibit - social, technological, and economic integration. Specifically, the inter- and intra-country equity in the region of the southeast Mediterranean is a serious issue in terms of both access and quality of the health services provided. Greece, Cyprus and Italy, as members of the European Union, have emphasized the sharing of eHealth services towards the provision of healthcare services equally to all the people, including people on the move. eHealth has lately been introduced and "*refers to the use of modern information and communication technologies (ICT) for enhancing health promotion and health protection, as well as quality, accessibility and efficiency in all aspects of healthcare delivery *[1]*"*. Recently, both at the national and European level, eHealth activities are being promoted as the means for citizen-centered health delivery systems with respect to the multi-cultural, multi-lingual healthcare traditions and diversities. This is in line with the report of the World Health Organization (WHO) Global Observatory for eHealth where the needs for electronic healthcare services in WHO member states are clearly presented [2].

Developments in telecommunication and computer science along with the availability of new and cheaper technologies have permitted the successful provision of eHealth services [3]. eHealth applications have been successfully used for the provision of healthcare services in rural health centers, in emergency cases, and for home monitoring of the elderly and people with chronic diseases.

Examples of successful applications of telemedicine for the provision of healthcare services in rural areas in developing and industrialized countries can be found in [4-7]. In [4] a novel ICT project in rural India is presented, where long-distance Wi-Fi network is used to enable high-quality videoconferencing between eye hospitals and remote village clinics, while in [5] the pilot program to implement telemedicine systems in rural sites in the center of the Peruvian Amazon region is presented. An empirical analysis of the consultation, information and training needs of health staff in rural areas of developing countries, to identify those needs that can be addressed by accessible communication networks [6], has shown that the key factor for a successful telemedicine system in rural areas of the developing world is its sustainability. Furthermore, in [7] a multipurpose healthcare telemedicine system is described. The system, designed for use in industrialized countries and operating via mobile communication network, satellite links or plain old telephone service (POTS), is able to provide a series of telemedicine applications that can be used for handling emergency cases in ambulances, in rural health centers, on ships, and for home telecare. Dedicated applications for the provision of telemedical services in vans [8], ambulances [9], ships [10] and aircraft [11] have also been presented in the literature.

ICT solutions have also been successfully used for enabling independent living for the elderly and patients with chronic diseases. Specifically, recent studies have shown that home monitoring of the elderly as well as of patients with chronic diseases increases the individual's comfort, enhances quality of life, and encourages patient empowerment, while it reduces the number of needless transfers at hospitals and the cost of provided healthcare services [12-16]. In addition, the use of technological and telecommunication infrastructures permitted the establishment of networks for both healthcare providers-to-patient interactions for outpatient consultation in remote underserved clinical sites [17,18], and healthcare providers-to-healthcare providers for telecollaboration [18,19], lifelong education [18] etc. Apart from the above, it has to be noted that recent studies have shown that eHealth solutions could be less cost-effective if benchmarked with other disease management programs not using ICT [20].

This paper presents a further step toward the provision of eHealth tools and services in isolated regions by creating a platform that tries to eliminate any limitations from communication and computational technologies and facilitates several tools and services independent of the case and place. The platform usage focuses on primary healthcare, patient home monitoring and physicians' continuous education. The study has been performed by a consortium (Greece, Cyprus, Italy) funded by the INTERREG IIIB ARCHIMED project "INTERMED: An INTEgrated broadband telecommunication pilot teleservices platform for improving healthcare provision in the Region of MEDiterranean" (June 2006-October 2008).

The INTERMED platform, which is able to cover a wide range of the services mentioned above, can be used to:

• Enhance the medical services provided by remote and/or isolated rural health centers, through the facilitation of collaboration between these medical centers and central hospitals.

• Facilitate the remote monitoring of "at-risk" citizens, whether located in their home environment, such as elderly citizens, patients with chronic diseases, and/or post-surgery patients, or involved in emergency outdoor incidents and in need of continuous monitoring by medical experts during transportation to the medical center, as in the case of road accidents and transportation via ambulances or emergency incidents in islands and transportation via ships and/or helicopters.

• Support medical experts during diagnostic procedures and facilitate the provision of more accurate diagnosis through collaboration between various medical centers and the exchange of "second opinions" between medical experts located in different geographical areas.

• Facilitate the Lifelong-Learning-Process (LLP) desired by the EU, through the preparation of learning courses and the conduction of seminars for medical personnel including medical experts, paramedics and healthcare personnel supporting the "at risk" citizens.

## Methods

Responding to the above needs, a platform based on several state-of-the-art systems and tools has been created. The technical goal was to achieve interoperability related to systems' technical characteristics, language and national issues.

### A. Pilot Sites

**Figure 1:**
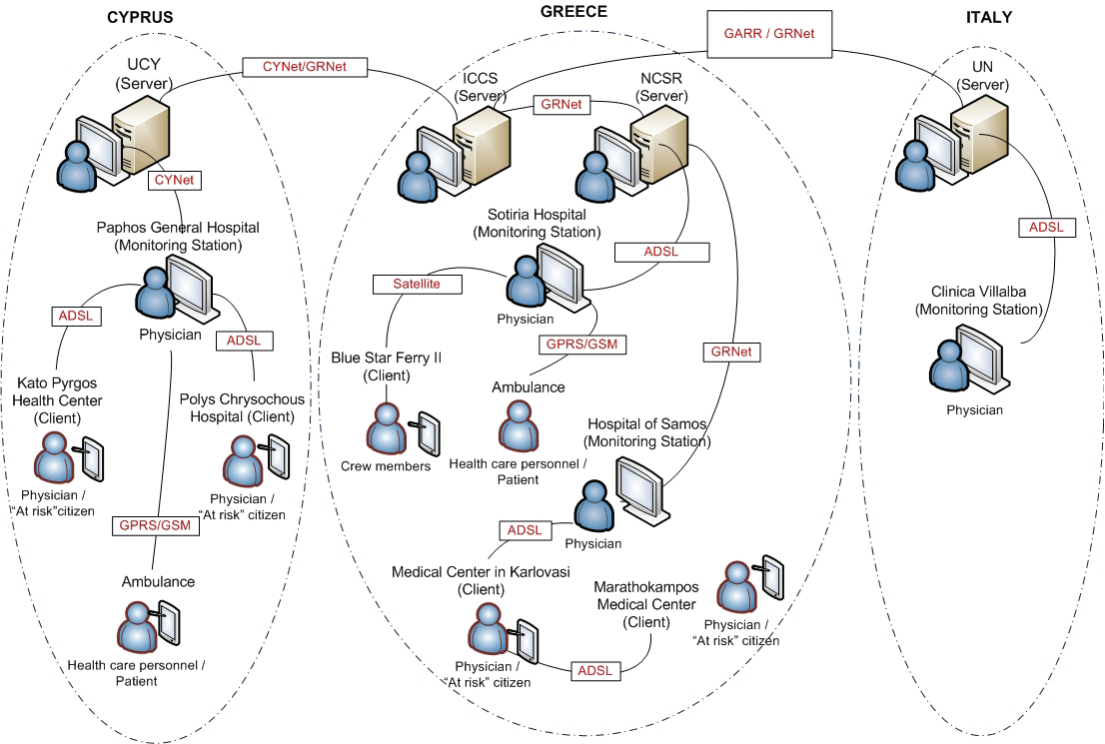
**Outline of INTERMED network**.

INTERMED aspired to facilitate the provision of telemedicine services at the point of need and aimed at evaluating different telemedicine systems at different pilot sites, each one with its own multidisciplinary needs and requirements. For this, the INTERMED platform was installed in various sites in Greece, Cyprus and Italy (named as end-points). From the total end-points involved in the platform (Figure 1), four were used as Servers, three as Monitoring Stations, and the remaining ten as Clients. More specifically:

• In Greece, the platform has been installed in the following ten end-points: Institute of Communication and Computer Systems - ICCS (Server), National Center for Scientific Research "Demokritos" - NCSR (Server), Sotiria Hospital (Monitoring Station), Medical Center in Karlovasi (Client), Hospital of Samos (Monitoring Station), Marathokampos Medical Center (Client), two "at risk" citizens' homes (Client), an ambulance (Client), and a Blue Star Ferry II travelling across the Aegean Sea (Piraeus-Rhodes, Client).

• In Cyprus, the platform has been installed in the following five end-points: University of Cyprus - UCY (Server), Paphos General Hospital (Monitoring Station), Kato Pyrgos Health Center (Client), Polis Chrysochous Hospital (Client), and one ambulance (Client).

• In Italy, the platform has been installed in Department of Electronic and Telecommunication Engineering of University of Naples Federico II - UN (Server) and in Clinica Villalba (Monitoring Station), a private hospital with a unit of cardiology.

All these seventeen end-points were involved in the pilot trials execution and the evaluation of the INTERMED platform. However, INTERMED did not regard the implementation of a single telemedicine system providing specific telemedicine services to a specific pilot site. On the contrary, as is explained in the next section, several telemedicine systems providing discrete telemedicine services were integrated into the holistic INTERMED platform. In this sense, not all telemedicine systems utilized within the context of the project were evaluated by each site. Rather, each telemedicine system was utilized by specific pilot sites either as a Client, or as a Server, or as a Monitoring Station.

### B. Services Description

The proposed system will be able to support primary healthcare, patient monitoring and physicians' continuous education through the combined use and mainly integration of already evaluated systems that were the results of several EU-funded projects.

#### 1) Teleconsultation Services

The teleconsultation services are mainly provided by the Wavelet based INteractive Video Communication System (WinVicos), which is a high-end, interactive video conference system [21]. Apart from the WinVicos, teleconsultation services are also provided by the systems used for telemedicine services (see next paragraph).

#### 2) Telemedicine Services

For both emergency telemedicine services and home care telemedicine services five different systems have been used.

The systems used for emergency and home care scenarios utilize state-of-the-art ICT and are capable of handling emergency medical incidents in random locations as well as emergencies inside healthcare environments. The integrated platform constitutes an innovative tool in the area of eHealth, and more specifically in the area of remote monitoring of patients. It comprises a combination of portable and/or fixed equipment that allows for the acquisition and transmission of diagnostically critical biosignals of the patient, such as various-lead electrocardiogram (ECG), blood pressure (BP) and oxygen saturation (SpO_2_), along with still images of the patient (upon which annotations can be made) and/or real-time audio-visual communication between the involved parties. Each of the used telemedicine systems is described in the following paragraphs:

• *Telemedicine System 1 (TS1)*: TS1 supports the aforementioned capabilities and is additionally scalable, in that appropriate device drivers can be developed for the collection of other biosignals such as blood glucose concentrations from glucose measurement devices. The TS1's architecture abandons the typical client-server architecture employed by the majority of telemonitoring systems that have been presented in the literature so far, and introduces the notion of nodes. There are three main participating nodes in each session, which can increment in its time-course. The "Transmission Node", which constitutes the mobile unit at the patient's site, is responsible for the acquisition of data from the medical devices and their local display, and triggers the request for the initialization of a medical monitoring session in order to transmit the data to specialized medical personnel. The "Monitoring Node", which constitutes the base station unit at the healthcare professional's site, is responsible for the reception of the "Transmission Node" request for monitoring, for the collection of the transmitted data, and for the provision of teleconsultation and telediagnosis. Last but certainly not least, the "Administrator Node" is responsible for the monitoring of all nodes and for the dispatching of monitoring requests from "Transmission Nodes" to "Monitoring Nodes", operating in automatic, semi-automatic or manual mode.

As mentioned earlier, TS1 supports the collection of various critical biosignals from standardized medical monitors, while several extension points exist so that plug-ins can easily extend the core functionality of the platform. Each device that can be viewed as a biosignal source - whether it is another medical monitor or any other measuring device - is uniquely related to a device driver, which enables the communication between the device and the TS1. Standard medical monitors, as the one utilized during the pilot trials, are capable of recording the waveforms and arithmetic values of the patient's 3 to 12 lead ECG, heart rate (HR), non-invasive blood pressure (NIBP), SpO_2_, temperature (TEMP) and respiratory rate (RESP). One of the main innovations of the system is the collaboration feature, meaning that a "Monitoring Node" can make an invitation to another node, to acquire what is called a "second expert opinion". In this context, the second expert receives the same information (biosignals and images/video of the patient) as the originally invited expert node, and can assist the first expert in the evaluation of the clinical case. TS1 also supports other audio-visual communication among the participating nodes. Thus, it supports the audio communication between the transmission node and the monitoring node utilizing Voice over IP technologies (VoIP), as well as the transmission of still images from all participating nodes, compressed by default utilizing the JPEG standard, while most common image formats are also supported, such as BMP, TIFF and PNG. To utilize this feature, the user of the platform can either select an image locally stored or acquire one by a web camera attached to the PC, and remotely transmit it to the medical expert for evaluation. In addition, the system is capable of transmitting video among the participating nodes.

The system prototype is the result of two EU-funded projects named Emergency 112 [7] and AMBULANCE [9]. During INTERMED, the TS1 was utilized during several telemonitoring sessions between the Sotiria Hospital (Monitoring Station) and an ambulance (Client), as well as between the Hospital of Samos (Monitoring Station) and the Medical Center in Karlovasi (Client). It was also utilized as the main server node at ICCS during the exchange of EHR data between ICCS and NCSR as well as between ICCS and the server nodes in Napoli and Cyprus.

• *Telemedicine System 2 (TS2)*: The TS2 is used for the Cyprus case. This system is based on a centralized controlled client-server architecture [7], [9]. The system consists of i) the patient units, which are the units located near the patients and ii) a central unit called base station which is located at a central hospital.

These patient units can be portable or not depending on the use case (ambulance, rural health center, navigating ship), while they are responsible for collecting and transmitting in real time several biosignals and images of the patient to the central unit. Each unit consists of a biosignals acquisition device, an image acquisition device, a communication device and the central processing device (personal computer).

○ The biosignals acquisition device is the device responsible for collecting several biosignals from the patient, like 3 to 12 lead ECG, SpO_2_, HR, NIBP, invasive blood pressure (IBP), TEMP and RESP.

○ The image acquisition device is responsible for collecting images of the patient that help doctors have visual contact with the patient.

○ The communication device is responsible for controlling the communications with the central unit. This can be a GSM/GPRS/UMTS modem, a satellite modem or a network communication device if it is not on a portable setup.

○ The central processing device is actually a personal computer, netbook, laptop or desktop, depending on the case. This device facilitates the software that controls real time acquisition of biosignals and images and real time transmission to the central unit. Furthermore, the software supports automated continuous communication of the telemedicine units with the central unit; this means that a connection will be automatically re-established in case of connection failure. The connection and communication procedures are all automated on the site of the telemedicine unit so as not to disturb the paramedics or any other personnel handling the patient.

The central unit consists of a dedicated workstation (personal computer) which facilitates the software for receiving all data coming from the telemedicine units. On this site, the expert doctor is able to see in real time biosignals and images of the patient. The telemedicine unit can be remotely controlled by asking for images or by changing the kind of biosignals received. In general, this unit allows the doctor to be visually present near the patient and give proper instructions to the non-specialized personnel handling the patient.

**Figure 2:**
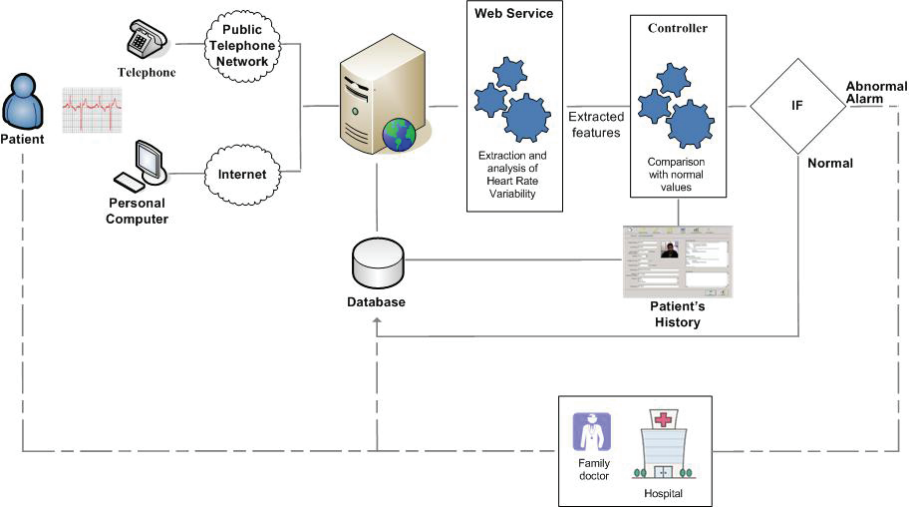
**Overview of services provided by TS5**. Dashed line corresponds to data and solid to communication.

• *Telemedicine System 3 (TS3)*: The TS3 is used mainly for the provision of home care telemedical services in the Samos Island. It consists of components that permit the acquisition and transmission of the patient's biosignals and weight. TS3 has been presented in detail in [22].

• *Telemedicine System 4 (TS4)*: TS4 is used for emergency telemedicine services onboard Blue Star Ferry II. The service is provided using the TraumaStation system [23] and covers telecardiology, ultrasound examination and videoconference for general consultations. The system provides a second opinion service for passengers and staff of the ferry. TraumaStation is a light, portable telemedical first-aid device that enables ultrasound imaging and acquisition of ECG, BP and SpO_2_signals all in a suitcase. Further, it offers tools for 2D/3D image acquisition and handling (enrichment of the images with graphical and textual annotations and pictograms) included the acquisition of 2D/3D ultrasound data and the management of images in DICOM format. In addition, the system is equipped with all available telecommunication gateways. Text messages and online-mode interactive communication via usage of a chat window are also available.

• *Telemedicine System 5 (TS5)*: TS5 installed in Italy i) is designed following a human-centered design approach [24] ii) is based on the client-server architecture iii) supports the above capabilities, and iv) introduces a node for the "remote processing" of the acquired biosignals. The additional node permits the analysis of ECG for the long-term home monitoring of patients suffering from congestive heart failure (CHF) [25], and more specifically the processing of heart rate variability (HRV) [26-29], in order to prevent more dangerous conditions where further re-hospitalization is needed. The procedure followed by a patient with the help of a relative or nurse is briefly presented in Figure 2. The patient sends his ECG via internet or via telephonic line, according to his technological skills. The ECG is stored into a DB, while a web service extracts and analyzes HRV. The extracted features and information regarding patient history are sent to a controller. If there are anomalies or abnormal trends, the system generates a warning to healthcare givers [30]. The extracted information is stored, updating the patient's record. In case of a warning, a nurse calls the patient to discuss his condition. The call aims to understand if the abnormality is due to worsening conditions or to daily inappropriate behaviors such as bad alimentations or stress. Often the clinician can stabilize the patient's health status by modulating the followed medication therapy. In case the patient's condition has become worse, ambulatory visits to a major hospital are organized. It has to be noted that "remote processing" improves cost-effectiveness of disease management by preventing emergencies [31]. Additionally, TS5 acts as a centralized controlled client-server architecture, but both client and server are independent nodes that use SOAP protocols to communicate between them and with the other nodes of the INTERMED subsystems when appropriate.

#### 3) eLearning Services

For the continuous training of the people who will use the platform, a series of distance learning courses has been prepared. The courses are provided through an appropriate website, where healthcare professionals and telemedicine experts have uploaded multimedia materials (audio and video files) and links to external knowledge sources. This material, which is compatible with the sharable content object reference model (SCORM) standard [32], can be accessed at any time and from anywhere. The service has been based on the use of the Moodle^© ^eLearning platform http://moodle.org/. The Moodle is an open learning management system (LMS) which permits the creation and organization of online courses and monitoring of the user's performance, and offers a series of tools for collaboration such as email, wikies, online forums, discussion boards and chats.

#### 4) Electronic Health Record

The electronic health record (EHR) created under the INTERMED platform is a longitudinal electronic record of patient health information generated by one or more encounters in any care delivery setting. Included in this information are patient demographics, progress notes, problems, medications, vital signs, past medical history, immunizations, laboratory data and radiology reports [33]. The EHR automates and streamlines the clinician's workflow, enabling real time and secure access to patient health record information whenever and wherever it is needed by the clinicians [34].

### C. System Integration

As mentioned earlier, it was the scope of the project not only to provide standalone telemedicine services at various sites, but also to validate the concept of eHealth. In order to achieve this, the project proposed a high-level integration of the described telemedicine services at EHR level. Namely, the project aspired to develop a common-reference medical record to which various telemedicine systems could store patient-specific data, and from which medical consultants could retrieve and evaluate patient-specific data (in the form of a short medical history) during or after a monitoring session, regardless of when and where other medical instances had occurred.

If, for example, a Greek citizen with a chronic condition that was being monitored at home using one of the telemedicine systems, travelled to Cyprus or Italy and happened to be involved in a car accident. If teleconsultation was required during the transportation to a hospital, the medical experts responsible for it could retrieve information from the patient's short EHR, regardless of the locality of acquired data or the system from which this data had been retrieved, thus achieving continuity in care.

It has however to be stressed at this point that it was out of the scope of the project to achieve integration and interoperability between the developed EHR and local healthcare systems, for a variety of reasons. The most important of these (leaving aside security and legal issues) is perhaps the (unfortunate) fact that even though most medical centers claim to support interoperability, the vast majority of them currently utilize closed-architecture systems based on proprietary protocols.

Nevertheless, even though information could not and was not shared with current EHR systems, the project tried to develop an open architecture that would facilitate future interconnection with other systems.

In this sense, connection to and interaction with the developed EHR was made feasible through the development of secure web services and the definition of solid "Get" and "Store" procedures for the retrieval and storage of data to the EHR respectively. For the deployment of the INTERMED common reference EHR, an XML schema that operates as an interface between different heterogeneous patient records was utilized. The use of the XML scheme facilitated the interoperability and interaction between geographically distributed systems, thus allowing the aspired patient mobility. The developed EHR was patient-centric, in that it followed a tree-structure with each patient constituting the root, which in turn could host several telemedicine sessions (branches). Each EHR session supports a fragmented message structure, with each fragment containing information regarding critical biosignals of the patients and their attributes, whether these were waveforms (i.e. ECG), arithmetic values (i.e. TEMP and/or BP) or images.

### D. Network Description

The services mentioned above are provided through a broadband telecommunication network that integrates three types of communication media: terrestrial, satellite and mobile (Figure 1). The network, which is based on the telecommunication infrastructure of the various end-points, provides coverage to all involved partners in Greece, Italy and Cyprus, ensuring communication between different and remote parties through data transmission and bidirectional communication by means of audio-visual content transmission. In the adopted topology, each end-point is connected to its appropriate router via an Ethernet link.

The overall networking infrastructure is divided into a core part and an access part. The core part of the network consists of i) terrestrial technologies of ADSL lines, the Greek research and technology network (GRNET) [35], the Cyprus research and academic network (CYNet) [36], and the Italian academic and research network (GARR) [37], ii) a satellite wide-area infrastructure and iii) a terrestrial wireless extension via 802.11 wireless protocol. The access part of the network consist of end-systems (server systems and user terminal) connected via fixed (Ethernet) or wireless (Wi-Fi 802.11) technologies to a local area network (LAN). The local LAN terminates with a router that connects the access network to the core network.

ADSL was selected to be the primary technology for use in the patients' premises as well as in the rural health and medical centers (such as Marathokampos Medical Center in Samos Island, Greece and Kato Pyrgos Health Center in Kato Pyrgos village, Cyprus). ADSL was chosen for the provision of i) home care telemedical services ii) teleconsultation and telecollaboration services and iii) telemedicine services in emergency situations, due to the low overall cost of usage compared to the other technologies available and the provision of high speeds needed for the provided services. Depending on the availability of ADSL lines, the patients and the health and medical centers are connected with speeds of 1.024 Mbps download and 512 kbps upload.

The GRNET, CYNet and GARR are used to interconnect the various academic (University of Cyprus, University of Naples Federico II) and research (ICCS, NCSR) partners (T1 connections). All nets fulfill the broadband network needs of the platform.

Satellite technology was considered only for the ferry due to the high cost involved in bandwidth rent and in some cases in equipment installation as well. The *very small aperture terminal *(VSAT) technology via which the ship communication (internet access) was established was the single channel per carrier (SCPC) technology. The network management has been undertaken by the EUTELSAT representative in Greece. A virtual private network (VPN) connection has been used to interconnect the application at the Blue Star Ferry II and the Sotiria Hospital. The VPN connection has been implemented using the OpenVPN [38] solution which is a full-featured SSL VPN solution able to accommodate a wide range of configurations, including remote access, site-to-site VPNs, Wi-Fi security, and enterprise-scale remote access solutions with load balancing, failover and fine-grained access-controls. A direct satellite link has been established through the Blue Star Ferry II and the satellite provider. The satellite provider was connected to the ferry company's headquarters through a terrestrial leased optical fiber line, and the company's headquarters was connected to NCSR's database via an ordinary internet connection using the VPN interface.

For the telecollaboration service, a 2 Mbps ADSL line between NCSR and Sotiria Hospital was re-activated and used. The Medical Center of Karlovasi was connected with the Hospital of Samos using ADSL. The physicians within the medical service of Karlovasi were using Wi-Fi (802.11.a) enabled PCs to connect to the medical center's router that terminated the ADSL. Mobile communication links (3G/GPRS) were used for the ambulance case in Cyprus. Mobile networks cover the whole area of Cyprus, enabling the connection of the ambulance vehicle to the Central Hospital for all cases.

A summary of the various services provided per pilot site and the utilized connections is presented in Table 1.

**Table 1:** Connected end-points, provided services per connection and transmitted data

Connected end-points	Connection utilized	Services	Data transferred between end-points
ICCS (Server) - NCSR (Server)	T1/T1	EHR	Incident data (patient info, ECG, vital signs)

NCSR (Server) - Sotiria Hospital (Monitoring Station)	T1/ADSL	EHR	Incident data (patient info, ECG, vital signs)

Sotiria Hospital (Monitoring Station) - Ambulance (Client)	ADSL/3G (fallback to GPRS when 3G not available)	Teleconsultation, Telemedicine	ECG, vital signs, images

Sotiria Hospital (Monitoring Station) - Blue Star Ferry II (Client)	ADSL/Satellite	Teleconsultation, Telemedicine, EHR	ECG, vital signs, ultrasound video, real time video conference

NCSR (Server) - Hospital of Samos (Client)	T1/T1	Teleconsultation, Telemedicine, eLearning, EHR	Incident data, patient info, ECG, vital signs, real time video conference

Hospital of Samos (Monitoring Station)- Medical Center in Karlovasi (Client)	T1/ADSL	Teleconsultation, Telemedicine, eLearning, EHR	Incident data, patient info, ECG, vital signs, real time video conference

Hospital of Samos (Monitoring Station) - "At risk" citizen (Client)	T1/3G (fallback to GPRS when 3G not available)	Teleconsultation, Telemedicine, eLearning, EHR	Incident data, patient info, ECG, vital signs, real time video conference

Medical Center in Karlovasi (Client) - Rural Medical Center in Marathokampos (Client)	ADSL/ADSL	Teleconsultation, Telemedicine, eLearning, EHR	Incident data, patient info, ECG, vital signs, real time video conference

Medical Center in Karlovasi (Client) - "At risk" citizen (Client)	ADSL/3G (fallback to GPRS when 3G not available)	Teleconsultation, Telemedicine, eLearning, EHR	Incident data, patient info, ECG, vital signs, real time video conference

ICCS (Server) - University of Cyprus (Server)	T1/T1	EHR	Incident data

University of Cyprus (Server) - Paphos General Hospital (Monitoring Station)	T1/ADSL	EHR	Incident data

Paphos General Hospital (Monitoring Station) - Kato Pyrgos Health Center (Client)	ADSL/ADSL	Teleconsultation, Telemedicine, eLearning, EHR	Incident data, patient info, ECG, vital signs, images

Paphos General Hospital (Monitoring Station) - Polis Chrysochous Hospital (Client)	ADSL/ADSL	Teleconsultation, Telemedicine, eLearning, EHR	Incident data, patient info, ECG, vital signs, images

Paphos General Hospital (Monitoring Station) - ambulance (Client)	ADSL/3G (fallback to GPRS when 3G not available)	Teleconsultation, Telemedicine	ECG, vital signs, images

ICCS (Server) - University of Naples (Server)	T1/T1	EHR	Incident data

University of Naples (Server)- Clinica Villalba (Monitoring Station)	T1/ADSL	Teleconsultation, Telemedicine, eLearning, EHR	Incident data, patient info, ECG, vital signs

## Results

The INTERMED project has implemented an end-to-end system for telehealth and teleeducation applications, integrating wireless broadband networks with terrestrial broadband technologies. The system's main purpose is to facilitate the sustainable provision of integrated eHealth and eLearning services and applications to an adequate number of distributed interconnected sites over a variety of telecommunication infrastructures.

The platform has been tested and validated in 17 pilot sites in Greece, Cyprus and Italy. It has to be noted that the platform and the provided services were assessed by citizens, travelers, patients, crew members and physicians in the sites mentioned above.

In the first publication of the INTERMED consortium, the initial architecture regarding involved sites, provided services and planned activities to meet the user requirements have been presented [39]. This paper describes the final architecture and design of the entire platform, the telecommunications infrastructure, the supported services, the tools and the system integrated. In addition, it presents the preliminary evaluation results, which are followed by future research directions and overall conclusions.

INTERMED, which is based on the integration of terrestrial, satellite and mobile networks, provides to the direct beneficiaries (residents, travelers, crew members, medical staff) the services described in the Methods section.

The final platform initially went through technical evaluation in order to ensure the correct and reliable operation of the several subsystems and the exchange of information between the subsystems from different countries. Following the technical evaluation, we tried to evaluate the acceptance of the final product from the users' point of view. This was done using questionnaires that helped investigate how the system can be used to improve the healthcare procedures followed in every use case.

During the pilot trials, four different use scenarios of the INTERMED platform have been evaluated. That is (a) home care (b) rural health center (c) ambulance/emergency and (d) navigating ship. For all cases, the evaluation was performed by all involved users (being either at the site accepting the consultation advice or at the site providing consultation). In tandem with this evaluation, participants were also asked to examine the eLearning tools offered as an infrastructural service to INTERMED users.

Keeping in mind that the INTERMED platform was using different subsystems, we had to create an evaluation scheme that could collect the results in a comparable way. In order to achieve this data was collected using two approaches:

• **Technical evaluation: **Including general results related to technical parameters of the systems collected during the pilot tests.

• **Users' satisfaction: **Including data collected from the final users based on their experience during the pilot tests.

### A. Technical Evaluation

In order to assess the technical performance, the INTERMED systems were evaluated as follows:

**Module Testing: **For this type of testing, each individual module was tested to determine whether all the system's modules are logically and functionally correct. If an error occurred, a suitable debugging procedure was followed.

**Integration Testing: **This form of testing was applied to all interfaces among modules to ensure that transfer of both data and control were performed correctly. This testing method included the run-time evaluation of all variables that are passed from one module to another.

**Function Testing: **The results and outcomes were evaluated after the execution of the program in which every functional menu selection had been activated systematically in turn.

**Final System Testing: **The objective of this type of testing was the full test of the entire system on completion of the system development period. In order to achieve this and get comparable results, the INTERMED subsystems were evaluated on a number of technical factors that were selected as the most important for the platform's correct operation. These factors, which monitor the system's correct operation and adequate quality of service (QoS), were chosen in order to cover the various scenarios. Each technical test was repeated five times for the duration of 15 minutes. The five tests for 3G network transmission were repeated twice: a) five tests with the transmitting station moving at an average speed of 40 kmph and b) five tests with the transmitting station stable in one place. Three healthcare professionals from each clinical pilot site conducted the clinical evaluation of the system from the expert side, while a varying number of "patient-test subjects" provided feedback regarding the user acceptance. Finally, the technical evaluation of the system was conducted by the personnel that took part in the actual implementation of the INTERMED system and its services. The results are shown in Table 2.

**Table 2:** Overview of technical metrics of INTERMED services

	Connection loss rate	Ease of data transmission and data exchange	Integrity of data transmission	Interference to other medical equipment	User requirements fulfillment
**Home care**	Low rate of failures (~< 2%)	No problems reported	Excellent	None	Users satisfied, amendments asked for

**Rural health center**	Low rate of failures (~< 2%)	No problems reported	Excellent	None	Users satisfied, amendments asked for

**Ambulance/emergency**	Low rate of failures (~10%)	No problems reported	Excellent	None	Users satisfied

**Navigating ship**	Low rate of failures (~5%)	Problems because of limited bandwidth	Problems with ultrasound & cardiographs	None	Users satisfied

In detail, the first technical factor is **connection loss rate**. This is defined as the rate of disruptions, when connected to several telemedicine systems related to the total number of connections. The overall scores were very good in this area, with a low failure rate of less than 2% in the "home care" and "rural health center" scenarios while, as expected, failures for "ambulance/emergency" had the highest failure rate of around 10% and "navigating ship" of around 5%.

**Ease of data transmission and data exchange **was the second evaluation factor. It accounts for the ease of data transmission of biosignals and all other necessary data (e.g. ultrasound video) for effective diagnosis over the INTERMED network, the ease of data entry by users into the INTERMED system, as well as the ease of data exchange between the several subsystems. Given the trial results in the above technical factor (connection loss rate), the outcome of **ease of data transmission **follows the same pattern. In the "home care" and "rural health center" scenarios there were no problems with ease of data transmission. During the "ambulance/emergency" scenario, no problems were observed for this parameter. There were some connectivity failures recorded during the trials - however they did not affect transmission of biosignals to the server. Data was transmitted successfully during each repetition of the test. During the "navigating ship" scenario, the main problem recorded in this factor is linked to the limited bandwidth of the satellite network deployed by the Blue Star Ferry II during the trials. The bandwidth was not adequate for the transmission of biosignals and ultrasound data. No problems were recorded regarding the exchange of data between the systems from different countries.

The third factor, **integrity of data transmission **examines a parameter of paramount importance in telemedicine as suggested by the relevant literature [40]. Anything transmitted during a telemedicine session must maintain its integrity in order to enable users to get a complete and precise image of the remotely located patient. During the several scenarios examined during the pilot trials, we had to monitor four types of data transmitted through the network:

• Biosignals such as ECG 3-12 leads and SpO_2 _waveform

• Numerical data like NIBP, HR, SpO_2_, RESP and TEMP

• Image files: Pictures of the patient (used for cases of external injuries) and medical images like X-rays

• Video-conferencing files: These were recordings automatically captured by the video-conferencing software

According to the data recorded by INTERMED servers for the "home care" and "rural health center" scenarios, in all four types of data there has been no problem with the **integrity of data transmitted**. The data was 100% accurate and there were no complaints from either consulting (server) or consulted (client) units. During the "ambulance" scenario, despite a couple of times when connectivity failed, data has been transmitted successfully. Users did not express any problems regarding the procedures of data transmission. On the contrary, they were quite satisfied with the interface and the sequence of data entry. Successful transmission signified high quality of the relevant data. The reasons for this high success rate are the same as the ones analyzed in the "home care" and "rural health center" scenarios. The "navigating ship" scenario entailed the transmission of data through a satellite network. The purpose was to simulate the conditions of teleconsultation when the ship is at large and see whether this type of data transmission presents a better alternative to the standard procedures of radio-communication. Unfortunately, this scenario produced some problems in the quality of data transmitted. According to the data recorded by the INTERMED server, the satellite connection was poor. In the majority of instances, the data recorded by the ultrasound and the portable devices could not be transmitted properly since the connection failed before data could be transferred into the server. Obviously, this resulted in partially transmitted data that was unreadable by the client. When the satellite connection failed, both ultrasound data and biosignals were not transmitted correctly and the physician at the main hospital could not assess the situation of the patient.

The fourth factor recorded during the pilot tests is **interference to other medical equipment**, where for all cases no interference effects were reported.

Finally, the fifth factor recorded is **user requirements fulfillment**. In general, users were satisfied with the final appearance of the systems. The systems were according to a detailed analysis of user requirements but, as expected, we had several requests for minor amendments on the systems used in the "home care" and "rural health center" scenarios.

### B. Users' Satisfaction Evaluation

Following the technical evaluation, a small-scale qualitative evaluation of the systems was performed. The evaluation was focused on the usability and acceptance of the provided services by physicians, paramedics and "at-risk" citizens/carers. Whitten et al [41] point out that users see the telemedicine systems primarily as services provided to them and not as new technologies. With this in mind and the fact that the success of every system relies on user acceptance, user opinion and technical evaluation must be taken into serious account as well.

The users' satisfaction evaluation included collection of data on perceived benefits of the services provided. Specifically, the three types of users from both Monitoring Stations and Clients were given access for a limited period to the INTERMED systems in order to run the different scenarios. Then they were asked for narrative comments regarding their experience during the interaction with the system. Their views, from both Monitoring Station and client perspective, are summarized in Table 3.

**Table 3:** Perceived benefits of INTERMED services

Scenario	Type of users		
	
	Physicians	Paramedics	"At risk" citizens/carers
	- Good alternative for avoiding frequent visits to the hospital.		- Increased sense of security.
**Home care**	- Long-term appreciation of patient's situation.		- Avoidance of unnecessary visits to the hospital.
	- Useful in cases of emergency.		

	- Sense of "team" work.		
**Rural health center**	- Time savings from patients' transportation to large hospitals.		
	- Treatment of larger number of patients.		

	- Better alternative than not seeing patient at all.	- Improvement of treatment.	
**Ambulance/emergency**		- Increased sense of security.	

**Navigation ship**	- Better alternative than not monitoring/seeing patient at all.	- Improvement of treatment. - Increased sense of security.	

## Discussion

The evaluation showed that INTERMED services are generally reliable from a technical perspective. Technical problems were recorded mainly during the "navigating ship" scenario where signals and ultrasound video could not be easily transmitted due to bandwidth limitations. During the "ambulance" scenario, some problems with line failures due to the movement of the vehicle were reported, but this was expected and since the systems could revive quickly (in approx. 1.5 to 2 minutes), it did not cause problems. During the "home care" and "rural health center" scenarios there were no actual problems recorded. Although the user satisfaction was not systematically evaluated, the general interaction with INTERMED was characterized as a positive experience that makes them consider use of such a system in the future. The importance of qualitative and contextual parameters has been noted by both users and observers of the trials.

## Future Research

The future research direction is aiming towards the involvement of end users in the evaluation of the developed platform regarding usability and improvement of healthcare. The goal of this task will be to create systems that can be integrated to users' daily routine without disturbing or needing major changes. This will help the effort to persuade users about the usefulness of such systems. Further, emphasis will be given to the quality of life of individuals living or traveling in isolated regions. Other challenging research areas that the involved research groups are currently working on are related to the enhancement of the platform with intelligent features in terms of computational decision support.

## Conclusions

This paper presents a new concept of an integrated broadband communication platform that might be able to serve the population in hard-to-access areas of the southeast Mediterranean and could also be used on naval vessels without medical services on board. It utilizes terrestrial, mobile and satellite communication infrastructures ensuring bidirectional communication between specialized and non-specialized medical personnel or civilians. The platform has been installed in several sites in Greece, Cyprus and Italy. The overall architecture of the platform has been technically tested, while a series of scenarios has been evaluated. The evaluation results showed the feasibility of the INTERMED platform to be successfully used by physicians, paramedical personnel and civilians. Encouraged by these results, the optimization of the platform for a wider delivery of telemedical services in the near future is under consideration. What we can expect from such a platform is the improvement of healthcare services in remote areas through telemedicine and teleconsultation, which can lead to a reduction in costs by limiting medical transfers to an absolute minimum, reducing morbidity and mortality of patients, and increasing quality of life due to better quality healthcare.

## Competing interests

The authors declare that they have no competing interests.

## Authors' contributions

SGM and EK drafted the manuscript, KP and AA designed and developed the EHR, HP designed and carried out the study for the network, while all of them designed, developed and evaluated the services in Greece and Cyprus. RT, LP and UB designed and developed the services in Italy. GK supported drafting the manuscript. CP provided advice on the project and revised the draft manuscript. DK managed the project and revised the draft manuscript. All authors read, reviewed and approved the final manuscript.
